# Report of Committee on Practical Medicine

**Published:** 1858-11

**Authors:** 


(Continued from page 532).
as having occurred in the spring of 1856, was among those subjected to an antiphlogistic treatment, in which part antimony entered largely.

"Rheumatism is frequently met with, especially in the winter and spring months. It is introduced here simply to call your attention to Laennec's treatment of the acute and sub-acute varieties, by sedative doses of tartarized antimony. Although it is almost a specific treatment, it seems to be generally ignored by the profession. The antimony may be given as usually recommended in sthenic pneumonia, but I commonly administer it in doses of one grain every four hours. This dose is nearly always tolerated, nausea or vomiting at first, being the exception, not the rule. After a decided sedative impression is induced, the remedy must be discontinued a day, and then resumed at longer intervals. It will be necessary to guard the bowels with laudanum, if they are too freely acted on by the antimony. Bleeding may be dispensed with under this treatment. One who has never used the remedy in this way, will often be surprised to see how soon a patient with acute rheumatism will pass from a state of intense suffering to almost perfect ease, and the swelling and soreness rapidly disappearing, and the dry, parched and coated tongue becoming moist and clean.

"Neuralgia has become one of our most common diseases. It is seated most frequently in the head and face, but is often found in other parts of the body. It is usually intermittent, but sometimes remittent in character, and accompanied with slight febrile symptoms during the paroxysm. After the operation of a mercurial cathartic, twenty grains sul. quin. with twenty-five to thirty of carb. ferri, given in the intermissions, seldom fails to arrest the paroxysm promptly. In the remittent cases, these remedies have to be persevered in for a few days. Relapses are common, but they yield more readily than primary attacks. I have discarded local applications, but anodynes are sometimes necessary, also general bloodletting. Perhaps the only interesting feature in reference to this disease is its astonishing increase. Ten years ago it was rarely met with; now it is encountered almost weekly. I am sometimes asked whether it is not a new disease—something unheard of until recently."

Your chairman would beg leave to add some observations of a topographical character, in reference to that region of our State lying in the valley of the Des Plaines river, of which Joliet is the centre.

Having its source in Wisconsin, the Des Plaines runs within a few miles of the Chicago river, and flows on in a S. W. direction, until uniting with the Kankakee, about fifteen miles below Joliet, they form the Illinois river.

The valley of the Des Plaines as it passes through Will County, varies in width, being in some places quite an expanse of marshy prairie, and in other having only a narrow passage between the bluff on each side.

The stream is mostly rapid, and in some places easily forded in low water, having either a gravelly or rocky bottom. On the west side, for the most part of the way, there is a bluff of limestone rock; but on the east, the elevation of the land, before reaching the ordinary prairie level, is more gradual.

At an early day, the inhabitants suffered much from the ordinary miasmatic diseases. The first settlements were made near the river, and in the groves. Since the completion of the Illinois and Michigan canal, which was in 1847, the health of this region has been annually improving, from the fact, in part, of the drainage consequent upon such an improvement. An instance may be cited where such a result obtains, in the city of Joliet, where the canal is made by walling the banks of the river, with sufficient fall within the city limits to require two locks.

To give an account of the diseases that have prevailed within our knowledge in this vicinity during the last year, will require but little time. The year 1857 was the most healthy of any since the first settlement of the country.

In January and February, there were some cases of a continued type of fever which had prevailed during the autumn previous, and of which mention is made in the report in practical medicine, for the last year. There were frequent cases that were protracted for some weeks, and one in particular occurred in the person of a young lady who was a subject of angina pectoris. Having come to the brink of the grave in spite of starvation, and the potent effect of the most active

Hahnemanic treatment, she was happily restored by the use of stimulants, tonics and nutritious diet.

During the months of March and April, there were frequent cases of pneumonia, both sthenic and asthenic, and characterized by many of the morbid appearances seen in the typhoid fevers of the previous autumn. In May, June and July, there was but little sickness of any description, except occasional cases of our ordinary vernal intermittents or remittents.

In August and September, there were some cases of dysentery. A small proportion assumed a low typhoid grade, and proved fatal. The cases that came under my notice, were ushered in with a chill, which was soon followed by reaction, and voiding bloody slimy stools, with severe tenesmus. The duration was from ten to twenty-one days, according to the severity, and the length of time which had elapsed before calling medical aid. Many cases were similar to ordinary attacks of intermittent, with the dysenteric symptoms superadded. If the patient could be seen soon after the first chill, a few powders, containing each from three to five grains sul. quinine, with about the same amount of hyd. c. creta or powdered blue mass, and opium or Dover's powder in a dose sufficient to allay pain, would frequently stop the disease at once. But if the patient had complained for a day or two, or had taken a cathartic, the disease was very sure to continue for some days. When the disease was not cut short at once, the careful use of alterative doses of hyd. c. creta or blue mass, combined with an opiate, was followed with good effects.

The tenesmus was best relieved by the administration of enemata of from one to two ounces cold water, to which forty to sixty drops tr. opii had been added, with ten to twenty grains tannin, in about fifteen minutes after each stool.

It is usual to direct the use of injections *immediately after* the stools have passed, but there is so great irritability of the mucous membrane of the rectum that the enema will pass immediately away, without causing any relief.

But if sufficient time is allowed for the local irritation to subside, and the patient to get a little rest, much better effects will be realized.

Pain and tenesmus are the most important items in the train of symptoms in dysentery, and if they can be controlled, a great point is gained. Any one who has suffered personally with this distressing disease, can appreciate the extreme exhaustion attending the attempts to evacuate the bowels, where there is nothing to pass off, and the desire proceeds only from the condition of the mucous membrane. It seems reasonable that the anodyne course is imperatively called for, and should be followed up closely, while other remedies more directly constitutional in their effects are employed to correct the condition of the system. The avoiding of cathartic remedies in dysentery, we consider very important. It may be admissible on the first day after the attack, but generally the alimentary canal is sufficiently evacuated, without harassing the patient with medicines which serve to increase the irritability. In this, as in many other diseases, rest is very requisite.

In the books, laxatives and cathartics are generally advised. We find, too, that many advise the alternation of laxatives with opiates. They give opium, for instance, until the patient is comparatively free from pain, and the discharges have ceased; and for fear that constipation should result, a dose must be given to move the bowels, when all the distressing symptoms will return. We repeat it, then, that the use of cathartics, as they are employed by many, is cruel, as well as unscientific.

Allow us to mention, by way of illustration, some cases that occurred in one family, in which dysentery prevailed. First, a lady about 50 years of age, was attacked with the usual symptoms of dysentery, slight at first, and so much so, as to delay for two or three days in applying for medical advice.

The disease became seated, and assuming a low asthenic type, continued about twenty-one days. The principal treatment was opium or morphine, given in doses and at intervals sufficient to quiet pain and produce sleep. Turpentine emulsion was given with tr. valerian in equal parts, three times a day. Enemata of water, in which ℨss. tr. opii, and grs. x. tannic acid were added, given as occasion required. No laxatives were given at all, and absolute rest enjoined. The case resulted favorably, and the patient was not very much debilitated. Animal broth, well salted, constituted the principal item of diet and drink. Another member of the family, fifteen months old, was sick about twelve days, and was treated with opium and quinine given regularly and without intermission during the whole time, without a particle of anything to act upon the bowels, except, occasionally, a small quantity of hyd. c. creta at first, to act upon the biliary organs.

While the individuals above mentioned were sick, both parents of the little girl began to complain of chilliness, with uneasiness in the bowels, and slight dysenteric discharges.

Instead of administering a cathartic to remove any vitiated secretions which might be supposed to exist, a prescription like the following was given:

| | | |
|---|---|---|
| ℞ | Sul. quinine, | ℈j. |
| | Blue mass in powder, | grs. xv. |
| | Pulv. opii, | " v. |

Make five powders, and take one every three hours.

After taking the above powders, all the symptoms subsided. In seven days, however, the symptoms returned, but were met in the same manner, with a like result. There were numerous cases like these, and all were as easily cured by similar means, if taken soon enough.

But if, instead of this abortive treatment, a mercurial cathartic had been given, followed by castor oil or sul. magnesia, the chances for making a long bill would have been good.

Dr. Edmund Andrews, of Chicago, writes:

"In dysentery, I recommend an injection, as follows—

| | | |
|---|---|---|
| ℞ | Water, | Oj. |
| | Alum, | grs. xxx. |
| | Tannin, | " xv. |

Fiat. Solution to be used as, and adjuvant to, the ordinary treatment. One or two large injections of this kind generally puts an end to the bloody discharge *at once;* and the constringing effect on the vessels very materially abates the inflammation. It should be repeated every time there is any reappearance of blood in the stools. The solution should be made weaker in cases of children. In the most desperate cases the practitioner may control the effusion of blood with great certainty; though if the case is complicated with typhoid fever, the mere relief of the inflammation of the colon, accomplished by the injection, does not always suffice to save life. In such cases, the symptoms of typhoid fever still remain after the dysenteric symptoms have disappeared. The principle of this treatment is not presented here as at all new, but as showing that many physicians are too negligent of the fact, that the inner surface of the colon is as perfectly accessible to topical applications as the external skin, and that a more free use of astringent injections, is requisite than is practised by many."

Before leaving this subject, we will add that the lady referred to as laboring under dysentery, had recently come from Connecticut, whereas the rest of the family had been in the State more than a year. There was a peculiar condition of the nervous system, which was not noticed in the others.

It was subsequently ascertained that a severe and fatal form of dysentery appeared in her former place of residence about the time she was sick. She had been in this State but four or five weeks at the time of the attack, and it is probable her symptoms were modified by the predisposition acquired before coming West. Such instances are common, and it is well to inquire when we meet with disease in those recently arrived, as to the general topography of the region. An individual coming from a mountainous locality, might require even venesection and other depletive measures, which would be inadmissible in an old resident.

Under improvements effected in the management of individual diseases, we will begin with glycerine.

Dr. Andrews, of Chicago, recommends glycerine as a solvent for vaccine virus, and as a preservative of the same.

Prof. N. S. Davis, in the *Journal* for February, 1857, gives the following formula, to be used in cases of tubercular disease in its early stage, before the cough is accompanied by much expectoration:

| | | |
|---|---|---|
| ℞ | Glycerine, | ℥ij. |
| | Iod. potass, | ʒj. |
| | Sul. morph, | grs. ij. |

M. Give teaspoonful before each meal, and at bed time.

"If the disease is farther advanced," he says, "and expectoration more copious, we prefer the following:

| | | |
|---|---|---|
| ℞ | Glycerine, | ℥ij. |
| | Syr. iod. ferri, | ℥ss. |
| | Sul. morphine, | grs. ij. |

M. Teaspoonful every four or six hours."

Prof. D. commenced the use of glycerine in the treatment of phthisis three years since, in the manner above expressed, with satisfactory results.

This interesting substance was, until comparatively a short time since, used only as an external application. Being a solvent for numerous articles, it is very convenient, and its use is earnestly advised in all cases where there is a failure in any of the vital functions, resulting in debility and wasting of the system. We are using at this time, in a severe case of stomatitis materna, a combination of iod. potassæ and iod. ferri in glycerine, with good effects.

Prof. H. A. Johnson, of Chicago, recommends glycerine applied externally to the cutaneous surface in scarlatina, instead of oleaginous substances. He says that being a solvent for the secretions from the skin, it is greatly superior to oil, which tends to obstruct the natural action of the part. When the skin is dry, a moist and soft feeling is apparent in a few hours. It also relieves the intolerable itching attending the eruption. He also recommends its use in the affection better known by western people than in nosological works, viz, the Illinois scabies or "prairie itch." We have used it in the different hepatic affections with great benefit. For excoriated nipples, nothing is more satisfactory in its effects, than tannic acid dissolved in glycerine, applied immediately after the infant has nursed, and allowed to remain until the next time of taking the breast, when the part should be carefully washed. It is also recommended in the preparation of the different ointments, and but for the comparative expense would undoubtedly soon be substituted for the oily substances.

CHLORATE POTASH.

Dr. E. C. Ellet, of Bunker Hill, Ill., in the *N. W. Med. and Surg. Journal*, for March, 1857, recommends chl. potash in mercurial salivation, as a specific. In the adynamic type of fever, attended with fœtid breath, and dysphagia, he uses it with much benefit; also in sore mouth from various causes, which as results of any disease, debilitate the system. He details the case of his own child, five years of age, who was successfully treated by it while laboring under a low form of infantile remittent. This article was formerly used in cases supposed to result from want of oxygen in the system, but is not very generally used at the present time.

Prof. Davis, in Proceedings of Cook Co. Medical Society, published in the *Journal* for May, 1857, reports three cases of cardiac disease.

The first case was a patient laboring under acute rheumatism. Together with the usual local symptoms noticed in such cases, there was constant pain in the dorsal spine of a very severe character, with occasional paroxysms of severe pains in the lower extremities. The slightest motion, or even a moderate jar of the bedstead, would cause him to cry out with pain. The foregoing symptoms, with quick impulse to the heart's action, and a plain pericardial friction sound a little below, and between the nipple and sternum, were considered indicative not only of severe articular rheumatism, but an affection of the central portion of the spinal system of nerves, and active pericarditis.

After giving reasons for diagnosis, the Prof. considers the indications for treatment to be threefold, viz: 1st, To mitigate the sufferings of the patient by anodynes; 2nd, To arrest the inflammatory actions, and, 3rd, To prevent, or at least to limit very much, the resulting effusions.

To accomplish these purposes, the patient was directed to take the following:

| | |
|---|---|
| Sat'd tr. veratrum viride, | ℨj. |
| Vin. colchici, | ℥ss. |
| Tr. opii et camph., | ℥iss. |

Mix, and take one teaspoonful every four hours. Also,

℞ Hyd. sub. mur., grs. xij.

Pulv. Doveri, ℈ij.

Potass nitratis, ℈ij.

Mix, and divide into six powders; take one between each time of taking the veratrum viride mixture. In twenty-four hours after taking the above remedies, the febrile action was much reduced. The pulse from 110, and hard, was lowered to 80, and soft.

On the third day, conclusive evidences of effusion in the pericardial sac were presented. With the use of alterative doses of mercurials, with anodynes and sedatives, epispastics to chest and spine, and alkaline drinks, followed up with opium, quinine and nit. potass every night at bedtime, the external or articular pains had ceased; the indications of effusion had disappeared, and the modified *friction* sound was again audible.

In three weeks the patient was discharged.

The second case was, three years previous, a subject of articular rheumatism. Partial recovery took place, but for eighteen months previous to this time there were unequivocal signs of thickening of the valves of the aorta, with much hypertrophy of the ventricles.

Prof. D. gave the patient sat. tr. verat. viride, four drops with spirit nit. dulc. in water, every four hours, and three times a day one of the following pills, viz.:

℞ Acetas plumbi.

Pulv. digitalis fol. } aa. ℈j.

Pil. hydrarg.

M. F. Pil. xx.

Under this treatment the heart's action was much diminished, and the patient left the hospital much relieved, but with signs of valvular disease and hypertrophy.

The third case was one in which there were tubercular deposits in both lungs, with a large cavity at the apex of the left. Heart twice the normal size. Pericardium adherent almost entirely to heart, by old false membrane. Left ventricle dilated, and the walls thick, and both semilunar and mitral valves much thickened and almost of a cartilaginous hardness. Right ventricle of normal size and valves healthy, but the walls much thickened. The patient, while living under all these morbid changes, was much relieved by taking every four hours the following:

| | |
|---|---|
| Chloride sodium, | grs. xv. |
| Sul. quinine, | gr. j. |
| Morphine, | gr. ¼. |

We find, in the *Journal* for May, 1857, the Proceedings of the DeWitt County Medical Society.

The subject of typhoid fever was under discussion, and Dr. Noble gave the following case which came under his care:

"The patient was a man about fifty, of good constitution, did not get very *sick* at any one time for the first two weeks. The pulse, *during the whole period of the disease*, remaining below the healthy standard. At no time did it exceed sixty beats in a minute, most of the time only fifty. Dr. N. had known the patient for several years,—his pulse in health was seventy-five to eighty. The patient felt rather indisposed than sick—not sick enough to keep the bed. This state of things continued two weeks, when profuse hemorrhage from the bowels commenced, and he sunk rapidly. The Dr. treated him successfully with dilut. nit. acid."

We refer to the above case, because we have often met with cases of typhoid fever, not only of the mild grade, but also the more grave, when the pulse was morbidly slow.

When the pulse is as low as sixty or fifty in a minute, there is cause for apprehension, even in a case so mild, as to call for only the least amount of medication; for there must of necessity be some obscure cause operating to occasion a slow pulse. A similar remark may be made in regard to an intermitting pulse, or where the heart beats more than 140 or 150 in a minute.

Dr. Edmiston says he combines nitric acid with strychnia, as follows:

| | |
|---|---|
| ℞ Strychnia, | gr. j. |
| Nitric acid, | ʒj. |
| Tr. opii, | ʒij. |
| Aqua, | ℥ij. |

Dose not given, but a teaspoonful, we should judge, would be a dose. He says that with this mixture and turpentine emulsion, he had saved several patients who would have inevitably died.

In the December number of the *Chicago Journal* we find an article by Dr. F. W. White, upon the use of chloroform in paroxysmal typhoid fever. The patient was a lady twenty-five years of age, recently from New England. The symptoms as described by Dr. White, were those ordinarily met with in remittent fevers, and he accordingly prescribed the following, viz.:

| | | |
|---|---|---|
| ℞ | Hyd. sub. mur. | grs. xij. |
| | Pulv. Doveri, | ʒj. |
| | Sul. quinine, | ℈j. |

Mix. F. Pul. vj. One every four hours, alternated with spts. nit. dulc. The unfavorable symptoms continuing, on the second day fifteen drops chloroform were given with the spts. nitre, with some relief of the distressing symptoms. Being considered too irritating given in this form, the following emulsion was prescribed:

| | | | |
|---|---|---|---|
| ℞ | Pulv. acacia, | aa, | ʒiiss. |
| | Sach. albi, | " | |
| | Spts. nit. dulc., | " | ʒiss. |
| | Chloroform, | " | |
| | Aqua distill., | | ℥iss. |

Two teaspoonfuls every four hours, alternated with one of the following powders:

| | | | |
|---|---|---|---|
| ℞ | Pulv. opii, | aa, | grs. vj. |
| | Sul. quinine, | " | |
| | Sach. albi, | | ʒss. |

M. F. Pulv. vj.

On the fifth day, the symptoms were relieved somewhat; sixth day, still improved. This emulsion was continued about two days, when the unfavorable symptoms were relieved.

The reporter seems to credit the chloroform with cutting short the disease.

If the disease was a fixed typhoid fever, as we have seen that disease, it is doubtful whether any course of treatment would have cut it short, and if it was materially shortened by the chloroform or anything else, it must have been something else than the genuine typhoid fever.

We have frequently seen cases like the one described above, which were relieved by the use of different sedatives directed to the mucous system, but their action was considered as temporary in mitigation of symptoms that were only accidental, and not constituting the disease. But we forbear further remark, for fear of drifting upon the ocean of controversy in relation to the question, What is Typhoid Fever?

There is a series of articles in the different numbers of the *Journal*, of Dr. Edward Brown-Sequard's experimental and clinical researches applied to physiology and pathology, as applied to epilepsy. The details of his observations cannot be given in this report, but we would refer the members to the articles.

Any degree of success in throwing light upon this very interesting and, in most cases, incurable disease, should be hailed with joy by the medical profession. Epilepsy has always been considered in the light of an impregnable fortress, bidding defiance to any and all attempts at reduction.

Most of us have witnessed its effects upon the person of some esteemed friend, in whom, at first, the slightest variation from the normal standard was noticed, but still sufficient to excite alarm. That person would manifest slowly, but not the less sure, all the successive changes from bad to worse, until the intellect once vigorous, and the body robust, would waste away, showing, at one view, a mental wreck and a physical pile of ruins.

In the *Journal* for October, 1857, is an article by Dr. C. Angell, of Pittsburgh, Ind., in which he gives the result of two cases of tying one of the common carotid arteries for epilepsy.

The first in a young man of 20, who had had fits for three or four years, but increasing gradually in frequency and severity. The next day after the operation, the left side was paralyzed, with no relief of the other symptoms. He lived seven days, but had no fits.

The other case was in a man 40 years of age. The operation was performed on the 8th of July, and up to Sept. 18th following, had had only four fits, and could attend to his business—was better than he had been for three years.

### MILK-SICKNESS.

This form of disease is very interesting to the profession, especially in the localities where it prevails, and not the less so from the fact that there is a great deal of obscurity in reference to the primary cause. The text books have but little to say respecting it, for the reason that its prevalence is confined to certain localities instead of being general. There is wide difference of opinion among those who have observed its phenomena, as to what produces it. In the *Journal* for April, 1857, there are some interesting observations by G. W. Wilkinson, in an inaugural address.

He says: "The disease is excited by the eating of beef, milk, or butter from cattle that had eaten a mineral substance contained in the earth. His reasons for this supposition are, that it is most common, even where it is known to prevail, in dry seasons, when this substance is not washed off, or so much diluted as to be powerless. The water of some springs has been known to produce it, and if these springs are enclosed so that cattle have no access to them, the disease will disappear in the neighborhood."

In the article referred to, is an instance mentioned where the earth, in a certain place, was covered with a white mould, in spots not very near each other, and no larger than a dime. It appeared to have exuded from the earth; and pressed between the fingers, had more the substantial properties of a mineral than a mould.

The pathology and treatment of this disease is stated in an inaugural thesis in the *Journal* for January, 1857, and nothing said as to its supposed cause, except that it may be more like a vegetable alkaloid than of a mineral nature. Judging from the symptoms described by Mr. Phillips, the disease is one causing great depression of the vital energies, and undoubtedly produced by an extremely subtile poison. From the fact that there is torpidity of the bowels, with extreme nausea, there is an impression produced upon the nervous system which interferes decidedly with the healthy functions.

In the *Journal*, of June, 1857, is an article by Dr. John

Pickard, upon the pathology of milk-sickness in Parke Co., Indiana. Dr. P. takes the same view of that of Dr. Wilkinson, viz: that it is neither vegetable, animal, nor malarial. He cites an instance, as follows:

"A farmer, living about a mile from where the disease is known to exist, allowed his cattle to run out, but they never reached the infested district until the dew evaporated from the vegetation; the result was, his cattle nor his family were never attacked by the disease; were it a vegetable, the absence of the dew would not destroy its poisonous qualities."

Another family having suffered from its ravages, plowed up a pasture field, digged around the stumps, thoroughly turning all the soil, and sowed the field in grass, upon which they have kept their stock for twenty years, and at no time has milk-sickness made its appearance; while, on other portions of the farm uncultivated, it is as fatal as ever.

"From the observations and experiments," he says, "which have been made, where the citizens have suffered severely from the loss of property, and the lives of those still dearer to them, we deduce the following conclusions:

"1st. Milk-sickness is caused by a poison, and this poison is generated near the surface of the earth, from some peculiarity in the soil, or the soil and atmosphere combined.

"2nd. That the poison is a mineral or heavy gas, which appears to be produced in the night, and destroyed by the rays of the sun.

"3rd. A thorough cultivation of the soil will destroy it.

"4th. That it may be communicated from one animal to another by the flesh or milk. And,

"5th. That oleaginous substances act as a preventive, and will often cure animals affected with the disease.

"There is no difficulty in the diagnosis; the patient invariably complaining of great weakness and inability to perform labor. A little exercise produces trembling and fainting sensations. As the disease advances, vomiting and obstinate costiveness are invariable accompaniments, and during the whole time a peculiar odor pervades the room, unlike anything we ever met with.

"The treatment of milk-sickness is simple. We have previously stated that oleaginous substances are preventives, and we have found no remedies so efficient as castor oil, by the stomach if possible, if not, by enemata. Apply sinapisms to the stomach, give cooling mucilaginous drinks, overcome the costiveness as soon as the nature of the case will admit, and you have conquered the main obstacle to successful cure."

In the *Journal* of November, 1857, John C. Beck, of Cadiz, and J. W. Crook, of Rockport, Indiana, give it as their opinion that this disease is of vegetable origin.

Dr. Crook says that Dr. Drake was of the opinion that the disease was caused by the cattle eating the rhus toxicodendron, and that he is "strongly" of this opinion. Both pursue a stimulating course of treatment, and if these observations are correct, there is no doubt the system is affected in a manner similar to that in case of specific poisoning, whether animal, mineral, or vegetable.

Dr. J. P. Bruler, of Rockport, Indiana, in the May number of the *Chicago Medical Journal*, presents the view that it is a poison, which, being received into the system of an animal, may be communicated to another. After saying that it cannot be a poison of an ordinary character, he remarks: "But there is a large class of animal poisons, commonly called infections, which do possess the power of self-propagation, whenever they are placed in a proper condition for such development. One rabid dog could infect a score or more, and they, in turn, produce the disease in equal ratio. The same is true of small pox, measles, psora," etc.

Dr. B. considers it infusorial, and gives the following instance: "A gentleman of this town had a cow which ran at large, caught the disease, and gave it to her calf. The calf died, and his dogs ate its flesh; they, in a few days, sickened, with all the ordinary symptoms of tires, and both died. A pet crow was seen eating the dogs; in a few days it refused to eat, could scarcely be made to hop or fly, as was its custom when in health, and in a day or two died."

Here we have a case where the poison affected four animals, or classes of animals, and might have been further propagated, had the crow been eaten. The Dr. thinks the above facts refute the idea of its production by rhus toxicodendron, arsenic, or any known vegetable or mineral poison. Concerning the pathology of this disease, Dr. Bruler confesses himself ignorant. "The late Dr. S. P. Sisna, informed me that he had made *post mortem* examinations twice." In both cases were found the usual evidences of an active inflammation of the stomach, and particularly of the pyloric region. In one case the pyloric orifice, and most of the duodenum, were so thickened and contracted that a probe could scarcely be passed into the stomach.

We have dwelt more at length upon this subject, from the fact, that much obscurity and discrepancy of opinion exist among the profession as to its cause, and from all that can be gleaned, it still remains a dark subject.

Occurring as it does only in certain localities, it is incumbent upon those members of the profession who are residents of the places where it prevails, to investigate it, and communicate the results of their observations to the world. We have no decided personal opinion to express, having never seen a case of milk-sickness, but in what can be collected from the experience of others, there is one very important and prominent consideration connected with the subject. Whatever obscurity may attend its primary cause, it is a fixed fact in the minds of many, that it is communicable from one animal to another, by partaking of the flesh or milk.

We find no such phenomena attending the effects of any class of poisons, either vegetable or mineral. As has been previously remarked, there is a severe affection of the great nervous centres. Extreme debility, tremors, nausea and vomiting, and a suspension more or less complete of the peristaltic action, are found.

We have sometimes seen symptoms in a severe attack of vernal remittent, that were said by those who had resided in such districts to resemble those of milk-sickness. It may be modified, and aggravated by the influence of malaria. It is found most prevalent in a belt of territory comprising a portion of Ohio, Indiana and Illinois.

Dr. Bowen, of Wilmington, in Will Co., who has practised more than twenty years in this and the adjoining counties,

(To be continued.)

